# The Burden of Hospitalized Lower Respiratory Tract Infection due to Respiratory Syncytial Virus in Rural Thailand

**DOI:** 10.1371/journal.pone.0015098

**Published:** 2010-11-30

**Authors:** Alicia M. Fry, Malinee Chittaganpitch, Henry C. Baggett, Teresa C. T. Peret, Ryan K. Dare, Pongpun Sawatwong, Somsak Thamthitiwat, Peera Areerat, Wichai Sanasuttipun, Julie Fischer, Susan A. Maloney, Dean D. Erdman, Sonja J. Olsen

**Affiliations:** 1 Influenza Division, Centers for Disease Control and Prevention, Atlanta, Georgia, United States of America; 2 National Institute of Health, Thailand Ministry of Public Health, Nonthaburi, Thailand; 3 Division of Viral Diseases, Centers for Disease Control and Prevention, Atlanta, Georgia, United States of America; 4 International Emerging Infections Program, Thai MOPH-U.S.CDC Collaboration, Nonthaburi, Thailand; 5 Division of Emerging Infections and Surveillance Services, Centers for Disease Control and Prevention, Atlanta, Georgia, United States of America; The University of Hong Kong, Hong Kong

## Abstract

**Background:**

We describe the epidemiology of hospitalized RSV infections for all age groups from population-based surveillance in two rural provinces in Thailand.

**Methods:**

From September 1, 2003 through December 31, 2007, we enrolled hospitalized patients with acute lower respiratory tract illness, who had a chest radiograph ordered by the physician, from all hospitals in SaKaeo and Nakhom Phanom Provinces. We tested nasopharyngeal specimens for RSV with reverse transcriptase polymerase chain reaction (RT-PCR) assays and paired-sera from a subset of patients with IgG enzyme immunoassay. Rates were adjusted for enrollment.

**Results:**

Among 11,097 enrolled patients, 987 (8.9%) had RSV infection. Rates of hospitalized RSV infection overall (and radiographically-confirmed pneumonia) were highest among children aged <1 year: 1,067/100,000 (534/100,000 radiographically-confirmed pneumonia) and 1–4 year: 403/100,000 (222/100,000), but low among enrolled adults aged ≥65 years: 42/100,000. Age <1 year (adjusted odds ratio [aOR]  = 13.2, 95% confidence interval [CI] 7.7, 22.5) and 1–4 year (aOR = 8.3, 95% CI 5.0, 13.9) were independent predictors of hospitalized RSV infection.

**Conclusions:**

The incidence of hospitalized RSV lower respiratory tract illness among children <5 years was high in rural Thailand. Efforts to prevent RSV infection could substantially reduce the pneumonia burden in children aged <5 years.

## Introduction

Respiratory syncytial virus (RSV) is the most common cause of hospitalized respiratory illness among children <5 years of age in industrialized countries and a common pathogen identified among hospitalized children in developing countries[1], [2], [3], [4], [5].While RSV infection is also associated with a large burden of illness that can be managed in outpatient facilities [1], those that are severe enough to require hospitalization are most worrisome. Severe lower respiratory syndromes associated with RSV infection include pneumonia and bronchiolitis. Pneumonia is the leading cause of childhood mortality among children <5 years of age in all regions of the world, responsible for an estimated 19% of all deaths in this age group [6]. RSV has been reported to account for 5–40% of pediatric hospitalized pneumonia [7], [8], [9], [10].

In 2003, active surveillance for hospitalized pneumonia was initiated in two provinces in Thailand, Sa Kaeo and Nakhon Phanom provinces, with the goal of detecting and describing the burden and characteristics of pathogens causing pneumonia[11]. In Thailand, a middle income tropical country, information on disease burden is needed inform pneumonia treatment and prevention strategies. In addition, epidemiologic characteristics of pneumonia in Thailand may be applicable to other Asian tropical countries that lack the infrastructure for active case finding and cutting edge diagnostics. We describe the incidence, epidemiology, and clinical characteristics of patients hospitalized RSV infections, including radiographically-confirmed pneumonia, for all age groups from population-based surveillance in two rural provinces in Thailand during 2004 through 2007. In addition, we describe a new sensitive RSV real time reverse transcriptase polymerase chain reaction (rRT-PCR) assay for RSV.

## Methods

### RT-PCR Assays

RT-PCR assays used in this study to test for RSV included: 1) an automated fluorescent capillary electrophoresis-based RT-PCR (fceRT-PCR)[12] used to test samples collected prior to September 1, 2005; 2) a newly developed rRT-PCR described here used to test samples collected during subsequent years (see Text S1 for methods and validation); 3) an RT-PCR assay[13] used to distinguish RSV groups A and B in positive samples from Sa Kaeo Province from Sept 1, 2003 – Aug 31, 2005 (see Text S1). In addition, we tested for human metapneumovirus (HMPV), human parainfluenza viruses (HPIV) serotypes 1, 2, and 3, adenovirus, and influenza viruses type A and B using RT-PCR assays that will be described elsewhere [14].

### Study design, specimen collection, and data analysis

A detailed description of the population-based surveillance system for clinical pneumonia in all eight hospitals in Sa Kaeo Province and all 12 hospitals in Nakhon Phanom Province, Thailand has been published[15]. These two provinces represent typical rural provinces and border Cambodia and Laos. A study to determine etiologies among enrolled patients from the clinical pneumonia surveillance system began September 1, 2003 in Sa Kaeo and January 1, 2005 in Nakhon Phanom and has been described in detail[14]. Briefly, a patient was eligible for enrollment if he/she had at least one sign of acute infection (i.e., fever, abnormal white cell count) plus signs or symptoms of lower respiratory tract disease (i.e., cough, shortness of breath, etc), and the physician ordered a chest radiograph within 48 hours of admission. Each enrolled patient had a nasopharyngeal swab, acute and convalescent sera and clinical and demographic information collected. We considered repeat hospitalizations that occurred ≥14 days after the previous discharge date as individual hospitalizations. Chest radiographs were read by an expert panel and determined to be consistent with pneumonia. World Health standard criteria were used for children and adult criteria have been described previously [16].

Nasopharyngeal (NP) swabs in viral transport media and acute- and convalescent-phase serum specimens were collected, transported, processed and stored as described elsewhere[17]. TNA extracts from 200 uL of the swab specimens were prepared on the BioRobot MDx Workstation (Qiagen) and stored at −70°C or lower until testing by RT-PCR. Acute- and convalescent-phase sera were tested for RSV only during September 1, 2003 - August 31, 2005 on specimens from enrolled patients from SaKaeo Province by indirect IgG enzyme immunoassay as previously described[18]. A specimen was considered to have a positive viral result if RT-PCR testing was positive or if there was a four-fold or greater rise in antibody titer between the acute and convalescent sera.

We calculated crude and age-specific (<1, 1–4, 5–19, 20–49, 50–64, ≥65 years) incidence for RSV infections using population estimates from Thailand's National Economic and Social Development Board ^18^ for the period January 1, 2004 through December 31, 2007. Because not all patients eligible for our study chose to participate, we determined a crude incidence and an estimated adjusted incidence to account for RSV cases in eligible patients who did not enroll. For the adjusted incidence we assumed the proportion of RSV infections in the enrolled patients was the same as the proportion of infections among all eligible patients; the number of eligible pneumonia patients in each age group was multiplied by the age-specific proportion of RSV-infected patients and divided by the population estimates. RSV infections with other viral co-infections were included in incidence estimates; we assumed that RSV contributed to illness. Confidence intervals for adjusted incidence estimates were estimated using the 95% confidence intervals of the proportion of RSV positive tests assuming a binomial distribution.

We compared characteristics of patients with radiographically-confirmed pneumonia associated with RSV infections to patients with radiographically-confirmed pneumonia with negative RSV testing results. We also compared patients with RSV infection who had a chest radiograph that was not consistent pneumonia to RSV negative patients without pneumonia. Dichotomous variables were compared by chi-square test and included in an unconditional logistic regression model if p<0.1. Interaction terms and effect modification were ruled out. All analysis was performed in SAS (version 9.1, SAS institute, Cary, NC). Two-tailed P values <0.05 were considered statistically significant. The data set for this analysis was finalized September 2009.

All participants were informed of the study objectives and written consent was obtained. This study protocol was reviewed and approved by the Centers for Disease Control and Prevention (CDC) Internal Review Board and the Thailand Ministry of Public Health Internal Review Board.

## Results

### Study Results

During September 1, 2003 - December 31, 2007, 41,761 patient admissions were identified with acute lower respiratory tract disease in Sa Kaeo and Nakhon Phanom Provinces. Of these, 22,857 (55%) received a chest radiograph and were eligible for enrollment and 11,097 (49%) were enrolled. Among enrolled patients, 987 (8.9%) had a positive diagnostic test for RSV. During September 1, 2003 – August 31, 2005, when serology was performed on specimens from enrolled patients from Sa Kaeo Province, 44 (25%) of the 175 RSV infections were only detected by serology. Conversely, 62 (35%) RSV infections were detected only by RT-PCR and 54 (31%) were detected by both methods. The proportion of RSV infections only detected by serology was highest among older children and adults (≥65 years 4/8 [50%], 50–64 years: 1/1 [100%], 20–49 years: 3/10 [30%], 5–19 years: 8/15 [53%], 1–4 years: 24/102 [24%], <1 year: 4/39 [10%]).

**Figure 1:**
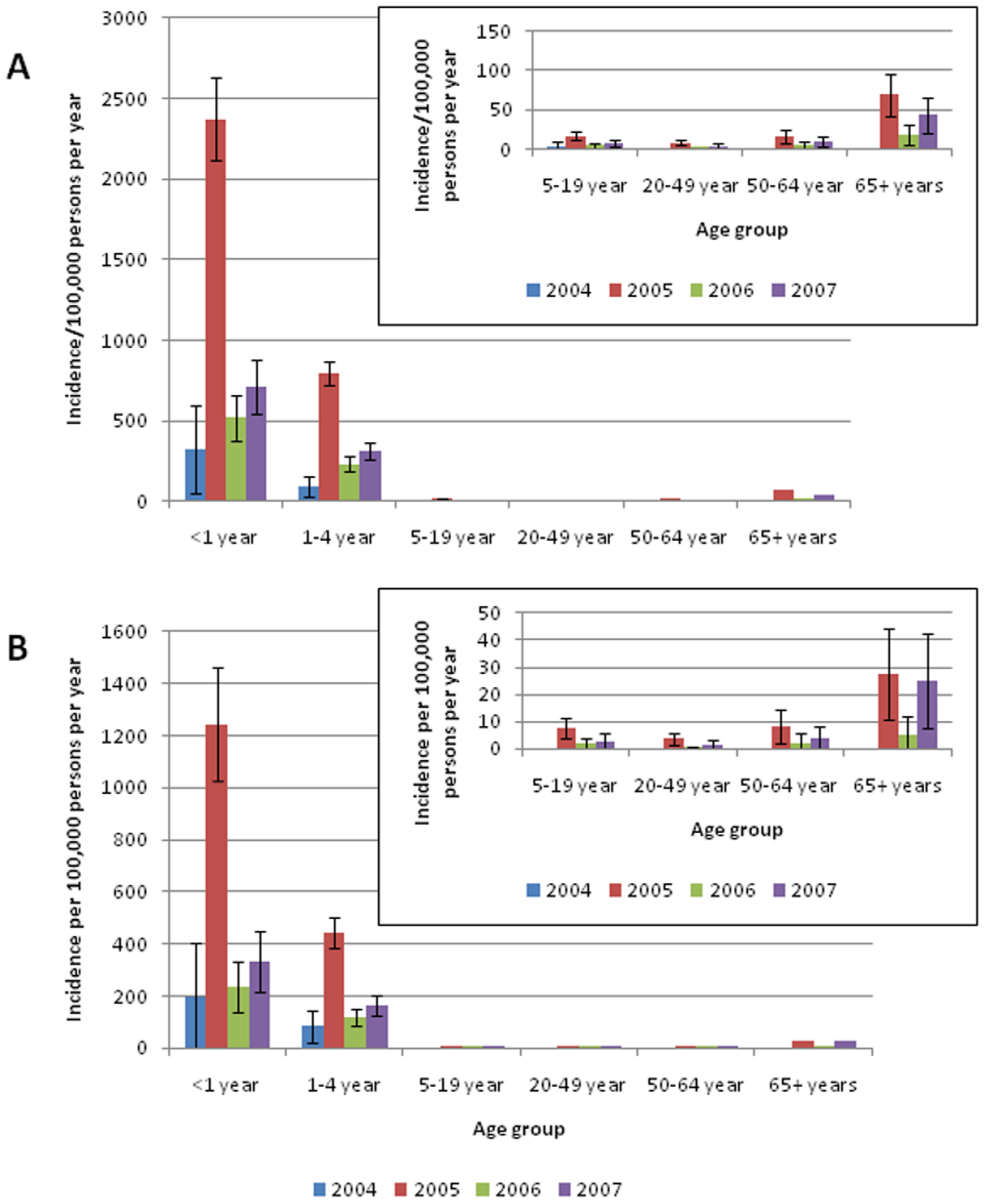
Adjusted annual incidence of hospitalized RSV respiratory illness (A) and hospitalized chest radiograph-confirmed RSV pneumonia (B) by age group, SaKaeo and Nakhon Phanom Provinces, during four consecutive years, January 1, 2004 – December 31, 2007. 2004 data are only from Sa Kaeo Province. Inset shows adjusted incidence values for age groups, 5–19 years, 20–49 years, 50–64 years, and ≥65 years.

Among all enrolled patients, most hospitalized RSV infections occurred among young children (Table 1, Figure 1). The highest incidence of hospitalized RSV-associated illness, including radiographically-confirmed pneumonia, was among children aged <1 year followed by children aged 1–4 years, a consistent finding each year during the four year period. The adjusted incidence of RSV illness among all enrolled children <5 years of age was 507/100,000 persons per year and the adjusted incidence of RSV pneumonia was 254/100,000 persons per year. Among enrolled hospitalized children with acute lower respiratory illness aged <1 year and from 1–4 years, 22% and 18% were associated with RSV infection, respectively. RSV accounted for 25% and 21% of all patients with radiographically-confirmed pneumonia among these two age groups, respectively. Radiographically- confirmed pneumonia accounted for 50% of RSV infections among enrolled children and 55% of all RSV infections among enrolled children aged 1–4 years of age. The incidence rates of RSV-associated illness were low for enrolled adults, including adults ≥65 years of age.

**Table 1:** Average crude and adjusted incidence for hospitalized patients with RSV infections, Sa Kaeo and Nakhon Phanom Provinces, January 1, 2004 – December 31, 2007.

						Incidence/100,000 persons per year		
Age group	Enrolled/Eligible No./No. (%)	Enrolled patients with RSV No. (% of enrolled)	Enrolled patients with CXR confirmed pneumonia No (% of enrolled)	CXR confirmed pneumonia with RSV No (% of patients with CXR pneumonia)	Crude incidence CXR confirmed RSV pneumonia*	Adjusted incidence CXR confirmed RSV pneumonia (95% CI)	Crude incidence RSV hospitalization among enrolled patients	Adjusted incidence RSV hospitalization among enrolled patients (95% CI)
1–11 mo	1313/2723 (49)	294 (22)	601 (46)	148 (25)	257	547 (464, 630)	524	1,087 (977, 1196)
1–4 yrs	2790/5250 (53)	505 (18)	1333 (48)	277 (21)	120	223 (198, 248)	216	406 (374, 438)
5–19 yrs	1069/1954 (55)	55 (5)	387 (36)	25 (6.5)	2.2	4.0 (2.4, 5.5)	4.8	8.7 (6.5, 11)
20–49 yrs	1616/3617 (45)	33 (2)	698 (43)	16 (2.3)	0.8	1.8 (0.9, 2.6)	1.6	3.6 (2.4, 4.9)
50–64 yrs	1509/3034 (50)	24 (2)	651 (43)	12 (1.8)	2.1	4.3 (1.9, 6.8)	4.3	8.6 (5.2, 12)
≥65 yrs	2577/5669 (45)	48 (2)	1189 (46)	20 (1.7)	7.5	16 (0.3, 24)	18	39 (28, 50)
Total	10,868/22,247 (49)	959 (9)	4850 (45)	498 (10)	12	24 (22, 26)	22	46 (43, 48)

CXR  =  chest radiograph, CI  =  confidence intervals.

* Among patients with radiographically-confirmed RSV pneumonia during January 1, 2004 through December 31, 2007, 46 had co-infections with other viruses, including one with HPIV1, five with HPIV3, 16 with adenovirus, 15 with influenza A or B viruses, and nine with HMPV. These patients were included in RSV incidence calculations.

Among enrolled children <5 years of age during 2004 though 2007, infection with RSV was most common among children aged 0–5 months (120/548 [22%]) and 6–11 months (167/741 [23%]). RSV infection was also common for each subsequent year of age, 12–23 months (251/1315 [19%]), 2 years (148/778 [19%]), 3 years (68/416 [16%]), and 4 years (36/272 [13%]) of age.

Independent risk factors for RSV infection among patients with radiographically-confirmed pneumonia included young age (Table 2). Compared to adults, children <12 months of age were 11-fold more likely to have RSV pneumonia versus pneumonia with a negative RSV test. Increased risk for RSV pneumonia was also notable for children 1–4 and 5–19 years of age. In addition, documented fever and tachypnea at admission were independent predictors for RSV infection among pneumonia patients. The month of patient admission was strongly predictive for RSV infection; admission during June through October was statistically associated with RSV pneumonia. We found similar risk factors for RSV lower respiratory infection with a chest radiograph that was not consistent pneumonia (RSV infection without pneumonia) compared to RSV negative patients without pneumonia, except for tachypnea.

**Table 2:** Independent predictors for RSV radiographically-confirmed pneumonia compared to non-RSV radiographically-confirmed pneumonia and for RSV respiratory illness without pneumonia compared to non-RSV respiratory illness without pneumonia, Sa Kaeo and Nakhon Phanom Provinces, September 1, 2003– December 31, 2007.

	RSV pneumonia vs. non-RSV pneumonia	RSV respiratory illness without pneumonia vs. non-RSV respiratory illness without pneumonia
	Adjusted OR (95% CI)	Adjusted OR (95% CI)
Age group0–11 months1–4 years5–19 years20–49 years50–64 years≥65 years	11.4 (6.6, 19.8)8.3 (4.9, 14.1)2.5 (1.3, 4.8)Referent0.8 (0.4, 1.8)0.8 (0.4, 1.7)	13.2 (7.7, 22.5)8.3 (5.0, 13.9)2.3 (1.2, 4.2)Referent0.9 (0.4, 1.9)1.3 (0.7, 2.4)
Temperature ≥38C	1.7 (1.3, 2.2)	2.6 (2.0, 3.3)
Tachypnea*	1.4 (1.1, 1.8)	1.1 (0.8, 1.5)
Wheezing	0.8 (0.6, 1.0)	1.0 (0.8, 1.2)
Month of admissionJanuaryFebruaryMarchAprilMayJuneJulyAugustSeptemberOctoberNovemberDecember	1.9 (0.6, 5.8)1.4 (0.5, 4.0)1.2 (0.4, 3.5)1.2 (0.4, 4.0)2.1 (0.7, 7.4)11.4 (4.4,29)21.3 (8.4, 53.8)12.0 (4.8, 30.5)9.4 (3.7, 23.8)4.7 (1.8, 12.4)2.4 (0.9, 7.1)Referent	1.2 (0.2, 7.1)2.7 (0.6, 12.6)1.9 (0.4, 9.2)3.4 (0.7, 16.9)4.4 (0.9, 20.3)13.1 (3.1, 55.0)37.2 (9.1, 153.3)27.7 (6.7, 114.3)16.7 (4.0, 68.8)11.3 (2.7, 47.6)5.3 (1.2, 23.2)Referent

* Age-specific tachypnea  =  ≥50/min if <12 months; ≥40/min if 1–4 years; >24/min if 5–9 years; >22/min if 10–14 years; >20/min if ≥15 years.

Among the 520 hospitalized patients with radiographically-confirmed RSV pneumonia, 67 (13%) had signs of consolidation, 413 (79%) had interstitial infiltrates, and 153 (29%) patients had alveolar infiltrates (no category was mutually exclusive).

Overall, 247 in-hospital deaths were reported for enrolled hospitalized patients during the four year study period; three deaths occurred among patients with RSV infection (one in a child aged 1–4 years and two in adults aged 20–49 year). Among patients with radiographically-confirmed pneumonia 179 (72%) deaths occurred, 83 (46%) among persons >65 years, 35 (20%) among persons 50–64 years, 46 (26%) among persons 20–29 years, 4 (2.2%) among persons 5–19 years, 6 (3.4%) among children 1–4 years and 5 (2.8%) among children <1 year. One of the 179 pneumonia deaths was associated with RSV infection in a 1-year old child. Two additional fatalities occurred among patients with RSV infection without radiographically-confirmed pneumonia, both were adults 20–49 years of age.

**Figure 2:**
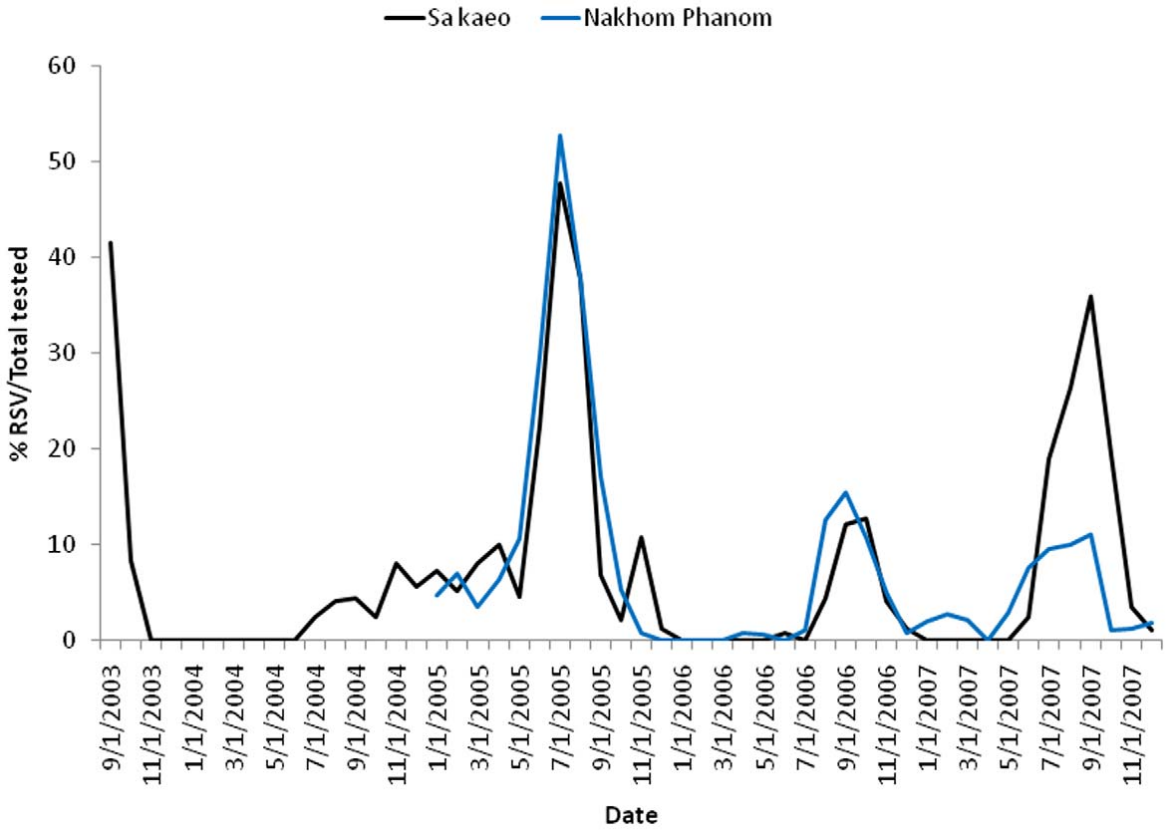
The percentage of patients with RSV infections per total patients tested by month and year among enrolled patients from SaKaeo (solid black line) and Nakhom Phanom (solid blue line) Provinces, September 1, 2003 – December 31, 2007.

During the period under surveillance, RSV circulation appeared to peak between June and October (Figure 2). During 2005, when annual RSV circulation was highest, RSV was detected for most months of the year but peaked between June and October. During July through October, RSV infection was detected in 2 to 47% of all enrolled hospitalized patients (all ages) each month. There was no difference in RSV circulation between the two provinces.

Among 225 available specimens with a positive RT-PCR test for RSV, 147 (65%) were group A, 49 (22%) were group B, and 29 (13%) had a negative typing test. During two RSV peak circulation periods, both group A and B RSV viruses circulated (data not shown).

There were no differences between patients with group A RSV infection versus group B infection with regard to fever (A:108 [78%] vs. B:13 [68%]), mechanical ventilation (A:0 vs. B:0), death (A:0 vs. B:0), or age-specific tachypnea (A:90 [98%] vs. B:12 [67%]). Patients with infection with group B RSV were more likely to present with wheezing (A:29 [21%] vs. B:10 [52%], Fisher's exact p = .008), were more commonly adults ≥65 years of age (A:2 [1.5%] vs. B:4 [21%]) and less commonly children aged 1–4 years (A:87 [63%] vs. B:6 [32%], X^2^ p = .004). There were no differences among the other age groups.

## Discussion

We demonstrated a substantial burden of RSV-associated pneumonia among children in two rural Provinces in Thailand during the study period; 1 in 187 children aged 1–11 months and 1 in 450 children aged 1–4 years were hospitalized annually for radiographically-confirmed RSV pneumonia. RSV accounted for 25% of hospitalized radiographically-confirmed pneumonia among children <1 year of age and 21% among children 1–4 years of age. In addition, RSV caused an almost equal number of additional hospitalizations among children for lower respiratory tract illnesses that were not radiographically-proven pneumonia. Age <1 year and 1–4 years were strong predictors of RSV-associated illness. In contrast, <2% of enrolled adults ≥65 years of age had RSV infection.

There are few population-based studies that report rates of RSV-associated radiographically-confirmed pneumonia. Our adjusted rate of RSV radiographically-confirmed pneumonia for all adults (3.6/100,000) was higher than that reported in the United States in the 1990s (1.6/100,000); however, we used a more sensitive assay, RT-PCR[19]. Rates of RSV-associated radiographically-confirmed pneumonia among children have not been reported from the U.S. or other industrial countries, or from developing countries. However, our rates of hospitalized lower respiratory illness (enrolled patients) were similar to rates of hospitalizations for the clinical syndrome of pneumonia and severe pneumonia in Kenyan children (children <1 year: 1107/100,00 and children <5 year: 293/100,000)[20]. Additional studies to document rates of RSV disease, including radiographically-confirmed pneumonia, in developing and industrial countries would put RSV respiratory disease and pneumonia into perspective with other commonly recognized causes of pneumonia. Given the recognized global burden of pneumonia, especially among children, the information from this study should inform planning for pneumonia intervention strategies.

To facilitate comparison of our rates with other reports that capture all hospitalized RSV infections (pneumonia, bronchiolitis, etc), we estimated the number of RSV acute lower respiratory tract infections that we missed by limiting our enrollment to patients who had a chest radiograph within 48 hours of admission and added these patients to our incidence estimates. The proportion of RSV infections among hospitalized children aged <5 years who met the case definition for acute lower respiratory tract illness but did not have a chest radiograph was 11% (HC Baggett, personal communication). Combining patients with and without a chest radiograph our estimated rate of hospitalized RSV acute lower respiratory tract infection among children <5 years would be 883/100,000. This crude estimation is consistent with rates for RSV-associated hospitalizations and severe disease among children aged <5 years reported in a recently published system review [5].

Prevention and treatment of childhood pneumonia are important components of the vision of the Global Action Plan for the Prevention and Control of Pneumonia (GAPP) [21]. However, at this time there is no effective and safe vaccine that prevents RSV infection in children and there are no proven treatment modalities. Available prophylactic measures against RSV, including palivizumab, are very expensive and only recommended for children at the highest risk for severe RSV disease[22]. New interventions to prevent and treat RSV illness could reduce hospitalizations for radiographically-confirmed pneumonia by up to 22% among Thai children <5 years of age and reduce at least a similar proportion of hospitalizations for other RSV lower respiratory tract illness.

We demonstrated annual peaks in RSV circulation occurring between July and October in Thailand. Patients admitted to a hospital with lower respiratory tract illness during these months were more likely to have RSV infection compared to patients with a negative RSV test. These peak months are similar to reports from other countries with tropical and subtropical climates[3] and other reports from Thailand[23] and correspond to the rainy season.

Unlike previous reports[24], [25], [26], we found no differences in severity of illness among patients with RSV group A and B infections. However, our analysis was limited by small numbers. We were unable to evaluate correlations between RSV load in nasopharyngeal specimens and severity, as reported by others [27]. We found more RSV group B infections among elderly adults than group A. Clinical studies from one candidate vaccine among the elderly, PFP-2 (purified fusion protein), found lower neutralizing titers to group B viruses compared to group A [28].

Our results are limited by some potential biases. We likely underestimated the incidence of hospitalized RSV infections. We did not enroll hospitalized patients who met the clinical case definition for acute lower respiratory illness but did not have a chest radiograph ordered within 48 hours of admission; we may have missed cases of bronchiolitis. Also, serology was performed for a limited time in one province but added a substantial number of cases during that time period. The lack of serology testing for the remainder of the study period likely reduced the number of RSV infections that we detected. This is especially true for older children and adults where approximately 50% of infections were only detected by serology when both RT-PCR and serology were performed. We did not adjust for the lack of serology in later years due to the small number of older children and adults who had both serology and RT-PCR performed. Very young children and very ill patients were less likely to enroll in the etiology study (H. Baggett, personal communication). Thus, we may have underestimated infections among children <6 months of age, and we likely underestimated the number of deaths, especially pediatric deaths.

The burden of hospitalized RSV-associated pneumonia and other RSV-associated lower respiratory tract illness among children <5 years was substantial in rural Thailand. Efforts to inexpensively and effectively prevent and treat RSV infection in children <5 years of age, including development of effective vaccines and treatment modalities could substantially reduce the global number of pediatric pneumonias and respiratory hospitalizations[21].

## Supporting Information

Text S1 (DOCX)
