# Supplementary material for: The Burden of Hospitalized Lower Respiratory Tract Infection due to Respiratory Syncytial Virus in Rural Thailand
Source: PLoS One. 2010 Nov 30;5(11):e15098. doi: 10.1371/journal.pone.0015098 (PMC2994907; doi:10.1371/journal.pone.0015098)
Supplement: Text S1 — (DOCX) [file pone.0015098.s001.docx]

**Supplement text for “**The Burden of Hospitalized Lower Respiratory Tract Infection due to Respiratory Syncytial Virus in Rural Thailand”

**Methods**

RSV rRT-PCR assay

The RSV rRT-PCR assay primers and probe were designed with the aid of Primer Express (Applied Biosystems) and Beacon Designer (Premier Biosoft International) to conserved regions of the matrix gene identified from alignments of sequences available in GenBank (M74568, M11486, AY911262, U39662, NC_001781, AF013254, AY353550). The primers and probe sequences include: forward primer

5’- GGCAAATATGGAAACATACGTGAA-3’, reverse primer

5’-TCTTTTTCTAGGACATTGTAYTGAACAG-3’ and probe

5’-CTGTGTATGTGGAGCCTTCGTGAAGCT-3’. Probes were labeled at the 5’ end with FAM and quenched at the 3’end with Black Hole Quencher-1 (Biosearch Technologies). Twenty-five-µL reaction mixtures containing 0.5 µM forward primer, 0.25 µM reverse primer and 0.05 µM probe, and 5 µL of specimen total nucleic acid (TNA) extract were prepared using the iScript One-Step RT-PCR Kit for Probes (Bio-Rad). Amplification was performed on an iCycler iQ^TM^ Real-Time Detection System (Bio-Rad) with the following cycling conditions: 48^o^C for 10 min (1 cycle); 95^o^C for 5 min (1 cycle); and 95^o^C for 15 s followed by 55^o^C for 1 min (45 cycles). Each specimen was also tested for the human ribonuclease P gene to insure TNA extract integrity.

The rRT-PCR assay was evaluated with RSV prototype strains Long (A) and 18357 (B) and multiple RSV field isolates representing genotypes GA1-7 and GB1-4[1]. Other respiratory viruses available for specificity testing included influenza viruses A and B, human parainfluenza viruses 1-4, human metapneumovirus, adenovirus, rhinovirus, human coronaviruses 229E and OC43 and human bocavirus. For limit of detection studies, RNA transcripts of RSV strains GA7 and GB3 were prepared from amplicons containing T7 and SP6 RNA polymerase promoter sequences generated using forward primer 5’-T7-GCGAAAAGACACCTCAGATGAAGT-3’ and reverse primer 5’-SP6-TGGAACATAGGCACCCATATTGT-3’ that bracket the region containing the rRT-PCR signature. RNA transcripts were synthesized, purified and quantified and replicate 10-fold serial dilutions prepared as previously described[2].

Distinguishing RSV group A and B

In addition, an RT-PCR assay was used to distinguish RSV groups A and B in RSV positive samples[3]. In brief, broadly-reactive primers complementary to a conserved region of the F protein gene, HRSVMPFO_2_ (5’-AACAGTTTAACATTACCAAGTGA-3’) and HRSVMPRW_2_ (5’-GCATCAATATCTCAAGTCAATGA-3’), were used to amplify a 357-bp product. PCR amplicons were sequenced and assigned to groups A or B based on alignments with representative RSV A and B F sequences, respectively.

**Results**

RSV rRT-PCR assay evaluation.

The RSV rRT-PCR assay amplified all prototype strains and representative genotypes of both groups A and B viruses. No false positives were obtained with a comprehensive collection of other respiratory viruses, human DNA or respiratory specimens confirmed negative for RSV by other methods. The assay reproducibly detected ≤ 50 transcript copies prepared from representative group A and B viruses, respectively. Reaction efficiencies, slopes and R2 values were 102.2, -3.27 and 0.995 for group A and 101.8, -3.28 and 0.996 for group B transcripts. The rRT-PCR assays showed comparable sensitivities with the fceRT-PCR assay using serially diluted RSV A and B TNA extract and identical results were obtained with 72 specimens positive for RSV by fceRT-PCR from the first study year. Thus, RSV detection rates were comparable with the two assays and can be combined for overall incidence calculations.

**References**

**1.** Peret TCT, Hall CB, Schnable KC, Golum JA, Anderson LJ. Circulation patterns of genetically distinct group A and B strains of human respiratory syncytial virus in a community. *Journal of General Virology.* 1998;79:2221-2229.

**2.** Dare RK, Fry AM, Chittaganpitch M, Sawanpanyalert P, Olsen SJ, et al. Human coronavirus infections in rural Thailand: a comprehensive study using real-time reverse-transcription polymerase chain reaction assays. *J Infect Dis.* Nov 1 2007;196(9):1321-1328.

**3.** Mazzulli T, Peret TCT, McGreer A, Cann D, MacDonald KS, et al. Molecular characterization of a nosocomial outbreak of human respiratory syncytial virus on an adult leukemia/lymphoma ward. *J Infect Dis.* 1999;180:1686-1689.
